# Bacteriophages: A Therapy Concept against Multi-Drug–Resistant Bacteria

**DOI:** 10.1089/sur.2018.184

**Published:** 2018-12-05

**Authors:** Christine Rohde, Johannes Wittmann, Elizabeth Kutter

**Affiliations:** ^1^ Leibniz Institute DSMZ-German Collection of Microorganisms and Cell Cultures, Braunschweig, Germany.; ^2^ The Evergreen State College, Olympia, Washington.

**Keywords:** antimicrobial drug resistance, bacteriophages, bacteriophage therapy, ESKAPE bacteria, microbiome, nosocomial infections

## Abstract

Bacteriophages (phages) are viruses that kill bacteria specifically but cannot infect other kinds of organisms. They have attracted new attention since the increasing antibiotic resistance developed into a global crisis. Phage therapy, a 100-year-old form of antibacterial treatment in medicine, is gaining momentum because phages represent a therapy concept without such negative side effects as toxicity; phages are the only therapeutic agent that regulates itself at the sites of infection and decays when the infectious bacteria have been killed. Nature is an almost infinite phage resource: New ones can be isolated for most kinds of problem bacteria as needed; bacteria and their phages constantly co-evolve. This is important as new pathogenic bacterial variants evolve and new challenging situations arise. In human therapy, “cocktails” of multiple phages may reduce the probability of selecting bacteria that developed resistance to a certain phage. Antibiotic agents can be applied together with phages in many circumstances; the two often function synergistically. Phages cannot be expected to replace antibiotic agents in our medical arsenal, but can be used where antibiotic agents fail. The selected phages, however, must be obligately virulent, well-characterized, and highly purified before application. Countless patients and their physicians are waiting for re-establishing phage therapy as a flexible, tailored medicine; infrastructures should be built in all countries urgently: The 2015 World Health Organization assembly resolution 68.7.3. called for national action plans by May 2017 to combat the antimicrobial drug resistance crisis. This article discusses the therapeutic potential of phages and describes challenges and recent developments.

## Antimicrobial Drug Resistance (AMR) Crisis: Can Medicine Still Rely on Antibiotic Agents?

For decades, beginning with penicillin, antibiotic agents completely transformed medicine and ended widespread disasters such as the Black Plague. Nobody expected that bacteria would successfully develop sophisticated, versatile defense mechanisms against all clinically relevant antibiotic agents [1,2], so that a post-antibiotic era would have to be declared by the World Health Organization (WHO) to be an alarming global threat [3]. Now, just a few years later, hundreds of thousands of deaths per year are caused by nosocomial infections alone, and the prognosis for the next years is much worse, as illustrated by economist Jim O'Neill (amr-review.org(2016)).

Already, nine years ago, more than 20.000 annual deaths in the United States were ascribed to the lack of successful antibiosis. For Europe, the situation is comparable. More than 100,000 persons worldwide died then from antibiotic-resistant bacterial infections annually. The situation gets worse: In France, about 13.000 people died in 2012 (www.invs.sante.fr), and worldwide, the current estimation is 700,000 annually.

In their 2016 General Assembly, the United Nations decided to work intensely on a plan for the development of new antimicrobial drugs and therapies on national, regional, and international levels to fight AMR, and national action plans were laid down in many Western countries. The European Union has established such a strategy within the Horizon 2020 program. The G7 Health Ministries called for a global “One Health” approach (www.g7germany.de) in 2015, and this was repeated during the G20 summit in Hamburg in 2017. Predictions are alarming, with 10 million persons estimated to die from AMR in 2050 if nothing changes [4,5].

Today, however, antibiotic agents that are important clinically are all only modifications of existing molecular structure principles; no true innovation is seen. Development and licensing of new antibacterial agents requires a long time, which is unacceptable in this global crisis. It is urgent to search for new compounds, but rapid resistance to new drugs is predictable [6]. Innovation must be “beyond borders, without geographical restrictions,” [7] as stated when the *CARB-X* initiative was introduced, focused very broadly on antibacterial products including any sort of therapeutics and on preventative measures.

The reason for the public health crisis of humankind is biologically simple: It is the ubiquitous abundance of bacteria, which are distributed by global circulation of air and water systems and also by all imaginable kinds of direct contact. Bacteria are globetrotters, and some of the pathogenic ones are robust persisters.

Today, the most formidable persister with clinical relevance is a new arrival to the general lists, *Acinetobacter baumannii*, which was placed number one on the WHO list of “priority one–critical” bacteria [8]. It is followed by long-challenging *Pseudomonas aeruginosa,* which is also extremely abundant and has enormous genetic flexibility and adaptability. They both easily move between natural and clinical environments and find their way into the human microbiome, perfect examples of the source-sink dynamics concept [9,10]. Both organisms are excellent colonizers, and often their colonization leads to actual infection; their flexible and versatile mechanisms of pathogenicity are unequaled.

AMR is distributed widely by horizontal gene transfer because of excessive use of antibiotic agents in human medicine and in much larger dimensions in animal farming, the latter creating a huge reservoir of AMR in combination with zoonoses. Unfortunately, major zoonotic bacteria belong to the ESKAPE group (see below), and these bacteria significantly contribute to the AMR crisis; they are living close with us, commensal opportunistic pathogens. The One-Health movement considers the triad including human, animal, and environmental health and has become of enormous importance in challenging the problems (www.onehealthinitiative.com) [11,12].

Bacteria carrying antibiotic resistance mechanisms bear a selective advantage, especially in habitats where this is particularly beneficial for them, such as clinical environments. Common opportunistic pathogens make up the ESKAPE set (Enterococci [vancomycin-resistant enterococci—VRE], *Staphylococcus aureus* [methicillin-resistant *S. aureus*—MRSA], *Klebsiella pneumoniae* [carbapenem resistant], *A. baumannii*, *P. aeruginosa*, and *Enterobacteriaceae* [extended-spectrum beta-lactamase—ESBL]), commuting between the natural environment and our human microbiome, and are the biggest threat because they exchange mobile genetic elements horizontally: Plasmids, temperate phages (see below), and transposons. Horizontal gene transfer happens also in the human microbiome [13], a rich and dense habitat.

Not only do antibiotic agents fail increasingly to be effective in human and veterinary medicine, but their use may also cause dysbiosis, especially in the gut or in secondary infections such as local or invasive candidiasis. Health consequences can be disastrous; e.g., after excessive, repeated antibiotic use has destroyed much of the natural gut flora, multi-drug–resistant strains of *Clostridium difficile* often colonize the gut and overgrow other micro-organisms [14].

Gram-positive bacteria such as MRSA or VRE were long the primary focus of the AMR problem, but now gram-negative bacteria that are resistant to three or four different antibiotic classes (3 multi-resistant gram negative [MRGN] and 4MRGN, respectively) prevail in nosocomial infections. An article published recently in a German medical journal stated that MRSA prevalence has been stable for some years, whereas the VRE prevalence increased by sixfold [15] (www.aerzteblatt-international.de). Both are on the WHO priority list for high need of action.

## There Is No Way Around the Microbiome and Its Phageome

Our healthy microbiome includes a virome that is, in fact, largely a phageome [16,17]. Most bacterial cells carry so-called temperate phages integrated into their chromosomes as prophages. These are silent inhabitants with a highly relevant regulative and co-evolutionary potential for our microbiota, but many of them confer antibiotic resistance and other unwanted gene sequences to their bacterial hosts, sequences that then behave like “jumping genes” when the prophages are released from their host genomes and enter a lytic cycle, free to infect other bacteria.

In contrast, obligately lytic phages are virulent and quickly kill their bacterial hosts by lysing them. As soon as they have adsorbed to the bacterial cell receptor, injected their nucleic acid into the bacterial cell, and begun to express their own genes, the host's imminent death is inevitable. The general consensus is that only these obligately lytic phages are appropriate for consideration for phage therapy applications, for a variety of reasons.

Lytic phages and prophages together are key in regulating global bacterial balances and also the healthy human and animal microbiome [18,19]. It can be assumed that our regular intake of phages from natural food and water resources is very substantial. This is a strong argument as to why allergies to pure phage preparations do not develop in humans, and why the innate immune system does not respond significantly to phages; it would disrupt the biological microbiome balance.

For *Bacillus* phages belonging to the SPbeta group, prophages have been reported to communicate via a phage-encoded peptide system appropriately called “arbitrium” that decides as a survival strategy determining lysogeny or the lytic cycle, a regulatory switch that is crucial because it ensures an existential balance for prophages to keep their bacterial hosts alive [20]. These findings seem so important that the “arbitrium” phage communication code seems to reflect a phage population dynamics system.

It is self-evident that such a dynamics control encoded by prophages plays a role also in our microbiome. Many prophages, however, encode and can transfer virulence factors and may thus be associated with pathogenicity, in a process called “lysogenic conversion”; this is very common in *Escherichia coli*, *Streptococcus pyogenes*, *Salmonella enterica*, and *S. aureus*. Prophages can, for example, encode exotoxins such as those causing the major pathogenicity of *E. coli* EHEC by inter-prophage interaction (verocytoxins or Shiga-toxins) [21] or of *Vibrio cholerae* (A-B-type exotoxin mediated by prophage CTX) [22]*,* as well as a rather broad spectrum of other proteins significant for bacterial virulence [23].

Because of the intensive relatedness to the human microbiome, most clinically important bacterial isolates, especially those of the ESKAPE group, contain both intact and residual partial prophage sequences in their genomes, which sometimes include these pathogenicity islands. It is our own dense gut microbiome habitat that inevitably causes constant genetic exchange and co-evolution of bacteria and their phages. There, the temperate phages actually outnumber the lytic, in contrast with most of the rest of nature, where virulent phages generally outnumber bacteria by an order of magnitude [24]. In this dense gut microbiome, it appears to be a selective advantage to carry prophages conferring virulence factors [25,26].

Extreme examples of prophage prevalence include the food pathogen *E. coli* EHEC O157:H7 strain Sakai, carrying 18 prophages, which amount to 16% of the total genome, and *S. pyogenes,* with up to six prophages as 12% of the bacterial genome [27,28]. Prophages have long-lasting bonds with their bacteria, a symbiosis that clearly supports the competitive position of both. The consensus among experts is that it would be extremely difficult to eliminate prophage gene sequences from such bacterial genomes.

The human microbiome has many very different compartments; most important in terms of surgical site treatment is the skin microbiome, where staphylococci are among the most frequent residents and which plays key health roles, so far under-investigated [29]. If the skin is injured, our own microbial inhabitants can invade, colonize, and cause infection. This may get dramatically dangerous if they are AMR and if a systemic infection develops. In the context of operations and surgical sites, MRSA has to be addressed specifically also because staphylococci tend to colonize on surfaces of implantation material and often cause a durable problem for a patient. There are already numerous animal models; a recent literature review sheds light on specific colonization and infection mechanisms, especially regarding *S. aureus* [30].

## Phage Therapy History until Today

Phages were used in human therapy soon after they had been independently reported by Frederick Twort in 1915 [31] in London and Félix d'Hérelle in 1917 at the Institut Pasteur in Paris. D'Hérelle applied phages first in 1919 in the Necker-Enfants Malades children's hospital in Paris, so phage therapy began in France, and explorations were continued in Europe and the United States through the 1940s; therapeutic phage cocktails were produced in Lyon and Paris until 1976. Felix d'Hérelle had collaborated with Georgian scientist Georgi Eliava in the early Institut Pasteur work and continued to correspond with him and perform phage work in Tbilisi, Georgia. The two finally founded the IBMV (Institute of Bacteriophage Microbiology and Virology) in 1933 in Tbilisi, which was gradually developed by their followers to be the world's biggest phage research and production center. Patient cases of earlier decades have been documented recently in English.

During the 2nd World War, particularly large volumes of phages were produced and used. The Institute became a branch of the Soviet Ministry of Health, and in the 1980s, 1000 employees were making over a ton a week of phage; 80% of it went to the Soviet military, mainly to combat the widely present dysentery. With the breakup of the former Soviet Union, the giant production center was privatized. Know-how has been kept diligently and farsightedly until today; volumes decreased, but tasks expanded and the broadness of projects is respectable, international reputation unquestionable [32]. In recent years, patients with AMR bacteria are traveling long distances to the close-by therapy center (eliavaphagetherapy.com) and an older phage therapy center (phagetherapy.com) to receive individualized phage treatment as a last hope. Patient cases of decades are documented [33], and recent case studies have also been published including a bilateral Georgian-German case study [34,35]. Clinical trials according to Western standards still have not been performed, however.

Also in Wroclaw, Poland, there is a renowned center for research into personalized phage therapy. Thousands of patient cases performed in local hospitals were documented to varying degrees, with healing rates generally corresponding with Georgian experiences. The Polish scientists around A. Górski (Ludwik Hirszfeld Institute of Immunology and Experimental Therapy) are well-known for their immunologic focus; they investigate immune response under phage therapy. In a study performed in 2008 to 2010 with 153 patients, no unwanted immune response could be described; phage efficacy seems independent of immune response [36–38].

While the Polish physicians apply exclusively monophage preparations, the Georgian doctors are applying mainly complex cocktails made by Eliava Biopreparations, although where they do not work, monophage preparations or individualized mixtures are prepared in the Eliava compounding pharmacy. Their major government approved classic cocktails have been evolved gradually since the 1930s: Intestiphage, targeting intestine-derived *Salmonella*, *Shigella*, *E. coli*, *Proteus*, *Pseudomonas*, *Enterococcus,* and *Staphylococcus,* and Pyophage for surgical site infections, targeting *Staphylococcus*, *Streptococcus*, *Pseudomonas*, *E. coli,* and *Proteus*. Both are tested and updated every six months, with phages added that target new problem bacteria, and are widely available in Georgian pharmacies. With aid from Europe, they are now moving toward Western Good Manufacturing Practice (GMP) standards.

Also, in Western countries, various trials including randomized ones, have been described since 2000 and, especially in Belgium and France, phages have been used in single cases of life-threatening infections according to the Helsinki Declaration, Art. 37. One example is the case of a patient with septicemia who had acute kidney injury [39]. Single patients have been treated with phages in the Queen Astrid Military Hospital, Brussels, where phage therapy can be described as being established.

A few European clinical trials have been run—e.g., phage therapy against *P. aeruginosa* in chronic otitis in London [40]; the biggest so far, “Phagoburn” (www.phagoburn.eu), a multi-center trial involving 11 clinical partners in Belgium, France, and Switzerland, focused on burn patients with topical use of phage cocktails against *E. coli* and *P. aeruginosa*. Phages are also prepared there for a multi-center trial targeting diabetic foot ulcers.

In Germany, “Phage4Cure” was launched in September 2017 with 100% government support to demonstrate the safety of highly purified phage preparations and finally reduce *P. aeruginosa* in chronically infected cystic fibrosis (CF) or non-CF bronchiectasis patients by phage inhalation; the clinical trial will be conducted in the Charité University Hospital, Berlin (http://phage4cure.de/en/). This clinical trial will be used to establish a GMP purification platform process for future phage preparations in Germany. Another clinical trial with focus on *P. aeruginosa* in CF patients has been launched by AmpliPhi Biosciences Corporation (http://www.ampliphibio.com); intermediate results have been published [41]. AmpliPhi is located in Australia and the United States and concentrates on phage production for therapy; besides *P. aeruginosa*, *S. aureus* is their top priority. Topical phage therapy is highly relevant also for treatment of diabetic ulcers; there are ambitious activities in the United States [42,43]— see also the *Wound Healing Society*.

For treatment of *S. aureus* surgical site infections, phage-containing patch-like matrices that continuously release phages to the areas have already been formulated [44]. In France, a research project on phage therapy in multi-drug-resistant *S. aureus* bone and joint infections and diabetic foot ulcers was launched in January 2015 (PHERECYDES; www.phosa.eu); see also www.clinicaltrials.gov. It was largely unknown until recently that in 1978 to 1985, phage therapy was applied in peri-prosthetic surgical procedures in the Helios Endo-clinic in Hamburg; more than 50 successful cases of phage use in advance of replacement operations after prosthesis infections are documented. Such infections are a torture, very difficult to treat, and characteristically carry biofilms.

## The Therapeutic Effect of Lytic Phages

Life inevitably involves biofilms! Micro-organisms live in complex communities and these make up our microbiomes, the richer the better for health (see above). Bacteria tend to settle on surfaces, layers of bacterial communities grow, and in medical terms, they often take on special relevance—e.g., in our oral cavities—in cases of implantation and chronic wounds, or in the lungs of CF patients. Could phages be an option to help degrade biofilms? Yes. Phages often penetrate biofilms better than antibiotic agents can, because of exopolymer-degrading enzymes such as polysaccharide depolymerases and by spreading through water channels to penetrate [45,46], whereas the efficiency of most antibiotic substances decreases through the layers of biofilms.

Studies with animals, humans, and tissue cultures have demonstrated that phages can enhance their efficacy by using mucosal tissue surfaces and at the same time cause a beneficial immune response: mucosa are the main entry points for bacterial infection, and it has been shown that phages are significantly abundant in mucus, with phages and mucus interacting via mucin glycans and Ig-like protein domains on some phage capsids [47]. Metagenomic analysis found indeed that these Ig-like proteins of phages are very common, especially in the vicinity of mucosa. This led to the “phage adherence to mucus model” that supports the idea of a co-evolution of phages and the eukaryotic bacterial hosts and a non-host-derived immunity. These pathbreaking results could shed light on a previously unknown antibacterial control of mucosa tissues [48].

This article deals with the use of whole natural phages, while another promising strategy uses various of the endolysins encoded by phages to break open the cell at the end of the infection cycle and engender release of the resultant progeny phages [49]. While in contrast to classic phage application, the application of the lytic enzymes means “static drugs”; the latter is definitely one of the two avenues for human medicine to manage bacterial infections with phages [50–54]. The lysin strategy is much more successful so far for gram-positives, with lysins from many different phages in late stages of development. In contrast, its implementation for gram-negative bacteria requires modification of the enzymes to allow the lysin to penetrate their characteristic outer membranes and reach the specific target sites in the peptidoglycan layer; research on this “artilysin” approach is now also far advanced (see Lisando GmbH, Regensburg, Germany) [55].

The most common phages in nature and those used for virtually all therapeutic applications are *Caudovirales*, with not only typical polygonal heads but also tail structures that mediate their binding to bacteria and transfer of their deoxyribonucleic acid (DNA) into the cell: Podoviruses, with short stubby tails; Siphoviruses, with long, usually flexible tails; and Myoviruses*,* where a similar long tail tube is encased in a sheath, contraction of which helps push the tail tip through the bacterial cell outer surface. Their ultrastructure, purity, and many questions on their interaction with host cells can be investigated only by electron microscopy. Each kind of tailed phage uses tail fibers to bind to highly specific receptors on particular kinds of bacteria, injects its linear double-stranded DNA molecule into the cell through the tail, in a process that requires cellular energy for most phages and, for lytic phages, immediately initiates an extensive re-programming of the host cell, which functions as a phage production factory. The details and extent of the reprogramming are highly varied for those relatively few phages where it has been studied extensively [56].

**FIG. 1.**
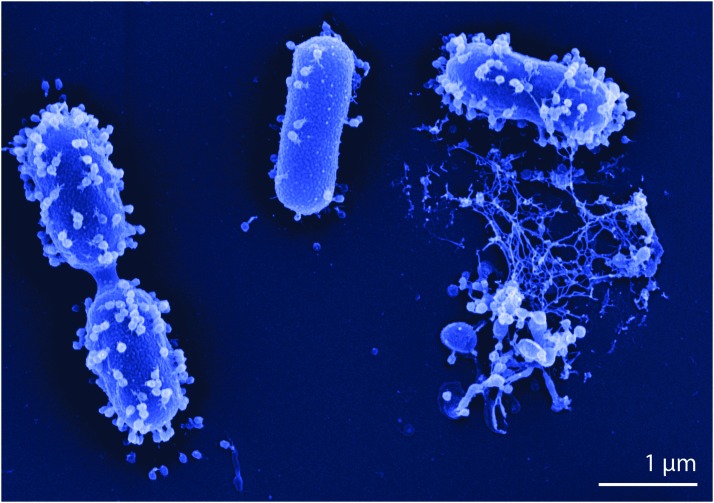
Phage vBAb-M-G7 on host strain *Acinetobacter baumannii* G7 and after host cell lysis. Scanning electron micrograph by Manfred Rohde, HZI, Braunschweig, Germany. Origin of phage and host strain: Eliava Institute IBMV, Tbilisi, Georgia.

At a pre-determined final time, the assembly of specific “holin” proteins forms a pore in the cell membrane through which their lytic enzyme molecules pass and poke a hole in the peptidoglycan layer, releasing a burst of new phages and other cell contents (Fig. 1). The process may take about half an hour at human body temperature and continues as long as suitable pools of host bacteria can be found—even if that involves being transported to other parts of the body in the blood stream or traversing the blood-brain barrier, as first demonstrated by Rene Dubow in 1943 and discussed in reviews such as [57]. In the human body, phages are partly or for the most part degraded by the reticular endothelial system [58,59], but they may survive for quite a while in sequestered places or even continue multiplying in reservoirs of their particular host bacteria, staying in balance with those bacteria as they do in the oceans.

Induced prophages occasionally transfer bacterial genes located close to their integration site in the bacterial genome, a process called “specialized transduction.” In addition, depending on their DNA packaging system, some temperate or even virulent phages will in rare cases package a phage DNA-sized piece of host DNA instead of their own, and thus randomly transduce a stretch of bacterial genes in a process called “generalized transduction”—a potential driving force of evolution. Obligately lytic phages cannot mutate to become temperate ones; this would mean substantial “gain-of-function” involving large sets of genes for integrases, recombinases, or repressors, etc.

Genome analyses and bio-informatic evaluation help decide about therapeutic suitability of phages and to exclude temperate ones. For a phage, AMR of its host bacterium is irrelevant, an appreciated advantage of phage therapy. AMR and bacterial phage resistance cannot be compared; these are completely different mechanisms. AMR is the globally manifest consequence of horizontal gene transfer whereas bacterial phage resistance occurs according to natural mutation rates in a bacterial population with different molecular background—e.g., receptor specificity or, after phage adsorption, mechanisms within the bacterial cell on the DNA or enzyme level [60]. For therapy purposes, phages with as low rates of bacterial resistance as possible should be selected, and phage mixtures (“cocktails”) could be used to minimize this problem.

Recent studies showed that by development of phage resistance because of receptor mutation, the susceptibility to antibiotic agents was restored again, suggesting that a positive synergistic effect of antibiotic agents and phages is possible [61]. Bacterial resistance to a phage is not a sustainable problem because new phages can be selected from natural samples. For the ESKAPE, the typical bacteria in nosocomial infections, in most cases new phages will be available readily and might be isolated from the patient's environment or from sampling directly the vicinity of the locus of infection.

Finding new phages for *S. aureus* is generally not trivial and means sampling the human and animal pharynx, nasal, and oral cavities. The *S. aureus* phages delineate a small, genetically uniform phage family typically with broad host spectra leading to effective bacterial coverages of very high percentage; the phage Sb-1 “family” is a prototype myovirus with Sb-1 being the first phage described for intravenous (IV) application in the Republic of Georgia. The Sb-1 and its relatives are components of the IBMV therapeutic phage preparations **[**62–64].

Since time immemorial, all life co-exists with phages so that unwanted side effects including toxicity or allergies are per se not likely. Strategies alternatively or additively to antibiotic drugs to combat the AMR crisis are claimed by doctors, patients, and scientists; phages do stand for the most logic alternative not only where antibiotic agents are ineffectivem but also in cases of their contraindication.

## Pharmaceutical Up-Scaled Purification of Phage Lysates

For the application of phages IV, the pharmaceutical phage production process and up-scaling must include a sequential purification so that the final preparation is free of growth media components, endotoxins (lipopolysaccharides), proteins, bacterial DNA, and exotoxins, and, of course, the phage titer (plaque-forming units per mL) must be sufficiently high. On the basis of all these considerations, international experts (P-H-A-G-E.org) have compiled a consensus scheme for the complete pharmaceutical production process [65–67], so that a model licensing pathway can be defined that will apply for future phage preparations and accepted by national authorities including the European Medicines Agency (EMA) in Europe. In June 2015, the EMA arranged a Workshop on the Therapeutic Use of Bacteriophages with approximately 60 stakeholders. Thereafter, experts compiled views with reference to this workshop [5,68].

Phages have average particle sizes of roughly 100–200 nm and are rather large compared with other medically active components, and they are the only medicine that needs bacteria for production. These two facts need an adapted licensing pathway requiring a dedicated production and purification process including specialized chromatography, filtration, and other techniques. Experts are discussing what optimal applicative phage doses should be—Georgian “cocktail” phages are successful but their titers are rather low; Georgian experience is undoubted, however.

The invaluable advantage of the specificity of phages is also a disadvantage: A suitable phage must be found/screened for an individual patient; a phage mixture could be an alternative in acute situations. Combining single phages into a “cocktail” is not trivial, however, because several parameters of phage biology have to be considered, and, finally, also the galenic ratio of the mixture. The larger a stock collection of purified phages for a certain bacterial species is, the better the chance to find suitable phages.

Modes of application frequency, duration of therapy, dosage, and pharmaceutical form have to be fine-tuned for each clinical trial or individual therapy. There seems potential to experiment with pharmaceutical forms of application because phages are stable as long as they are not kept at very acidic or basic pH value; they must not be frozen or heated or put under excessive shearing forces. The phage production strains must be chosen carefully and should be free of prophages before pharmaceutical production is started. This is not too challenging to investigate. Genomics gives the answer: Algorithms for finding prophage sequences are available, such as PhiSpy [69] or PHAST [70], and genome annotations elucidate the respective prophage properties [71]. A. Fauconnier [72] stated “Regulation needs to be adapted to phage therapy and not vice versa.” Neither the required infrastructures nor a harmonized licensing pathway exist in the Western world. Both are urgently required [73] because the applicative potential is broad [74].

## Development of Networks between Researchers, Companies, and Hospitals

Phage therapy has been applied empirically and often successfully for 100 years in various parts of the world and particularly developed in France, Georgia, Russia, and Poland. More tightly defined clinical trials in conformity with Western pharmaceutical standards and addressing different medical targets and bacterial pathogens are still needed now especially to establish efficacy and to support the urgent development of a model licensing pathway; basic safety for many purposes has been more established by the vast amount of use than any tightly designed clinical trial could. Some problems that arose for very few clinical trials have highlighted some of the major challenges in narrowly defined double blind studies, beyond the enormous expense and time delays that would be required to test even a fraction of the desperately needed applications in that fashion.

Recent developments in the United States involving the Food and Drug Administration or in Europe, including the European Medicines Agency, demonstrate that licensing authorities and health ministries strive for facilitating re-introduction of phage therapy. Infrastructures are already being developed in partnership between private and public sectors that support emergency phage application targeting ESKAPE pathogens, as demonstrated in the recent incredibly fast, highly publicized world-wide response to the case of collaborative rescue of the researcher Tom Patterson in San Diego who got a life-threatening infection with multi-resistant *A. baumannii* in Egypt.

As a result of such often untreatable *A. baumannii* infections found widely during the fighting in Iraq, medical branches of the US Army and Navy and the newly established phage therapy research facility at Texas A&M had all invested greatly in building up vast libraries of *A. baumannii* strains and isolating and cataloging a very large number of phages targeting this species, without knowing much about them. They had also taken advantage of the most sophisticated new approaches to design rapid often partly automated ways of fishing a needle out of a haystack and identifying those very few phages that could productively infect any given strain [75]. Excitingly, this has led rapidly to further patient treatments and the establishment of a hospital-associated phage therapy center there.

Two slightly different medium-term strategies have been developed: The “magistral” phage production [76], introduced in Belgium recently, and the sustainable large-scale long-term access supply inventory, the latter requiring phage banks with substantial holdings of purified or pre-purified phages, both finally tailor-made flexible medicine. The infrastructure that is needed includes networking phage banks that might be best located within established culture collections, GMP production facilities experienced in pharmaceutical phage purification, diagnostic units and medical doctors striving for this goal in hospitals. For true emergency applications with less common bacterial strains, compassionate use for individuals in hopeless situations should be possible. It seems crucial to include allowing immediate use of phages newly isolated with minimal regulatory requirements beyond those defining a complete production route. It is an additional advantage that phages and antibiotics are acting synergistically so that overnight phage screenings (phagograms) against patient isolates in parallel with antibiograms can produce results for tailored application.

Possible licensing pathways are being discussed and a model licensing pathway is needed urgently for preparations including phages as natural, specifically acting biological entities that are able to eliminate pathogenic bacteria and to self-regulate. Prudence and clinical/scientific control are important. Benevolently attending authorities do accompany the currently running clinical trials with goal-oriented questions, independently of intellectual property (IP) protection strategies. Pharmaceutically developed phage preparations are products, and it will be worthwhile to seek IP protection for certain larger-scale, more homogenous applications. IP may well be appropriate for specific use strategy, but not generally for individual phages themselves.

Governments should encourage and support approaches involved in developing appropriately designed clinical trials using phages therapeutically. In addition, more public and private money needs to be funneled into basic phage research, including details of the infection process under clinically relevant conditions for broad varieties of phages targeting ESKAPE pathogens. Fortunately, such phages can be isolated fairly readily. Thus, these most common key nosocomial pathogens can and should be targets of broadly collaborative, focused programs involving academics, corporations, and governmental agencies for re-introducing phage therapy in ways both ancient and innovative.
